# Quantum spin-valley Hall effect in AB-stacked bilayer silicene

**DOI:** 10.1038/s41598-019-55927-9

**Published:** 2019-12-19

**Authors:** Kyu Won Lee, Cheol Eui Lee

**Affiliations:** Department of Physics, Korea University, Seoul, 02841 Republic of Korea

**Keywords:** Nanoscience and technology, Physics

## Abstract

Our density functional theory calculations show that while AB-stacked bilayer silicene has a non-quantized spin-valley Chern number, there exist backscattering-free gapless edge states within the bulk gap, leading to a quantum spin-valley Hall effect. Using a tight-binding model for a honeycomb bilayer, we found that the interlayer potential difference and the staggered AB-sublattice potential lead to abrupt and gradual change of the valley Chern number from a quantized value to zero, respectively, while maintaining backscattering-free gapless edge states if the valley Chern number is not too close to zero. Under an inversion symmetry-breaking potential in the form of the staggered AB-sublattice potential, such as an antiferromagnetic order and a hexagonal diatomic sheet, a finite but non-quantized (spin-)valley Chern number can correspond to a quantum (spin-)valley Hall insulator.

## Introduction

Silicene, one of the two-dimensional group IV materials such as graphene, is a single layer of silicon atoms arranged in a honeycomb lattice and is similar to a buckled graphene lattice^1–4^. The low-energy physics of silicene can be described by the two-dimensional Dirac equation like graphene^1,4,5^. Silicene is a quantum spin Hall insulator with a spin-orbit coupling of 3.9 meV^1,2,5^, and various topological phases were predicted under an electric field and an exchange field^4,6^.

The layers in bilayer silicene can be bound together by covalent bonds unlike the layers in bilayer graphene bound by the van der Waals interaction. Due to a buckled structure, bilayer silicene can have various structures, which are semimetallic^7,8^. Multilayer silicene has been synthesized by several groups^9–11^, and effects of intercalation and hydrogenation on the (opto)electronic properties of bilayer silicene have been studied^8,12^. Most works on bilayer silicene have been done without considering electron-electron interactions. Under electron-electron interactions, bilayer silicene was predicted to be a topological superconductor^13^.

A planar AA-stacked bilayer silicene, where all the silicon atoms of a layer are covalently bonded to the silicon atom of the other layer, has the lowest energy^7^, but needs to overcome considerable energy barriers^14^. AB-stacked bilayer silicene, where the silicon atoms of a layer reside on top of a carbon atom (dimer sites) or a hollow center (nondimer sites) of the other layer, is known to be an antiferromagnetic insulator with an indirect band gap of 0.29 eV^14^. AB-stacked bilayer silicene has covalent interlayer bonds at the dimer sites, breaking the *π*-bond network of each layer, and the interactions of *π*-electrons localized on nondimer sites are responsible for the intralayer ferrimagnetic and interlayer antiferromagnetic order of AB-stacked bilayer silicene^14^.

On the other hand, the spin-valley degree of freedom characterized by the product of spin and valley indices is a new degree of freedom other than spin and valley, and can be employed for advanced electronics^4,15,16^. Inversion symmetry breaking together with spin-orbit coupling in monolayers of MoS_2_ was reported to lead to valley-contrasting spin splitting, which suppresses spin and valley relaxations and results in coexisting spin and valley Hall effects^15^. Antiferromagnetic order coupled to the valley degree of freedom was reported to result in the spin-valley degree of freedom, which leads to spin-valley-dependent optical selection rule and can lead to a renormalization of the valley gaps under a strong enough spin-orbit coupling^16^. In the *π*-band tight-binding models for monolayer and bilayer silicene, staggered exchange field was predicted to lead to a quantum spin-valley Hall insulator phase^4,17,18^.

The valley degree of freedom can be distinguished in systems where inversion symmetry is broken^19–23^. Under a small inversion symmetry-breaking potential, the Berry curvature is sharply peaked at each valley and the valley Chern number can be accurately defined^20,23^. If the inversion symmetry-breaking potential increases, the Berry curvature peak pertaining to each valley can be broadened and partially mixed with each other, and the valley Chern number obtained by integrating the the Berry curvature around a valley will deviate from the quantized value. In that case, it is not clear how the valley-related physics such as the bulk-edge correspondence would change. Actually, the inversion symmetry-breaking potential due to a spontaneous magnetic order may not be small enough to ensure the sharply peaked Berry curvature.

In this work, our density functional theory (DFT) calculations show that while the intralayer ferrimagnetic and interlayer antiferromagnetic order of AB-stacked bilayer silicene gives a non-quantized spin-valley Chern number, there exist backscattering-free gapless edge states corresponding to a quantum spin-valley Hall effect. Using a tight-binding (TB) model for a honeycomb bilayer, we found that the valley mixing occurs abruptly as the interlayer potential difference increases above a threshold, but occurs gradually as the staggered AB-sublattice potential increases. The interlayer potential difference induces an abrupt change of the valley Chern number from a quantized value to zero. The staggered AB-sublattice potential induces a gradual decrease of the valley Chern number from a quantized value, while maintaining backscattering-free gapless edge states corresponding to a quantum valley Hall effect. As a result, under an inversion symmetry-breaking potential in the form of the staggered AB-sublattice potential, such as an antiferromagnetic order and a hexagonal diatomic sheet, a finite but non-quantized (spin-)valley Chern number can correspond to a quantum (spin-)valley Hall insulator.

**Figure 1:**
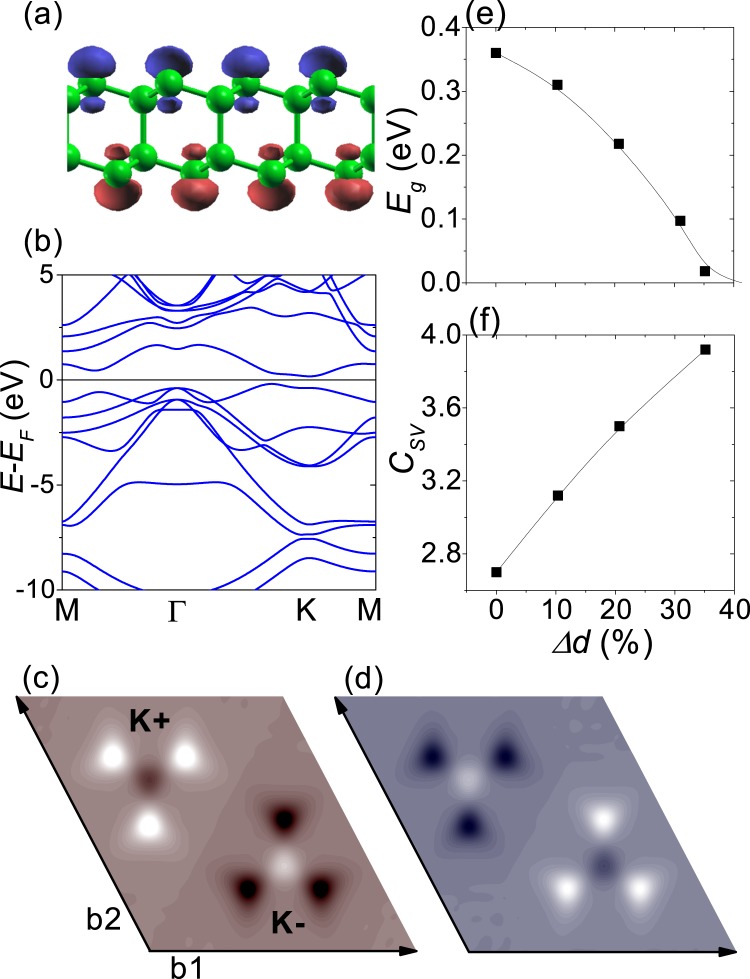
DFT calculations for bilayer silicene sheet. (**a**) Spin density. Green ball corresponds to silicon atom. The red and blue colors represent opposite spins. (**b**) Band structure. Energy bands with opposite spins were completely overlapped. (**c**,**d**) Show the Berry curvature maps for opposite spins. The bright and dark spots correspond to positive and negative Berry curvature, respectively. b1 and b2 represent reciprocal lattice vectors. K+ and K− correspond to the opposite valleys. (**e**,**f**) Show the band gap *E*_*g*_ and the spin-valley Chern number *C*_*SV*_ as a function of $\Delta d=(d-{d}_{0})/{d}_{0}\times 100$, where *d* (*d*_0_) is the strained (equilibrium) interlayer distance.

Figure 1(a) shows the spin density of AB-stacked bilayer silicene sheet. The spin density is mostly localized on the nondimer sites with intralayer ferrimagnetic and interlayer antiferromagnetic order as previously reported^14^. Figure 1(b) shows the band structure of AB-stacked bilayer silicene, where the energy bands with opposite spins were completely overlapped. The band structure indicates that the AB-stacked bilayer silicene is an antiferromagnetic insulator with an indirect band gap ~0.36 eV, consistently with a previous work^14^. Figure 1(c,d) show the Berry curvature maps for opposite spins. The Berry curvature shows opposite signs at opposite valleys and for opposite spins, indicating a possible spin-valley Hall effect. From the spin-valley-resolved Chern number $C(s,\xi )$ ~sign(*s*$\xi$)0.67, four types of the Chern number can be defined^4,18^. The Chern numbers were zero except for the spin-valley Chern number *C*_*sv*_ ~ 2.68, which is not quantized but still indicates a spin-valley Hall effect.

The Berry curvature shows broad peaks of small height. The Berry curvature peak pertaining to each valley can be partially mixed, giving the non-quantized spin-valley Chern number. A small inversion-symmetry breaking potential ensures the sharply peaked Berry curvature^20,23^, and the staggered magnetization in the AB-stacked bilayer silicene decreases with increasing interlayer distance^24^. Figure 1(e,f) show the band gap and the spin-valley Chern number as a function of $\Delta d=(d-{d}_{0})/{d}_{0}\times 100$, respectively, with a strained (equilibrium) interlayer distance *d* (*d*_0_).

The sublattice magnetization on the nondimer sites, obtained from the Mulliken population analysis, is 0.3 *μ*_*B*_ at equilibrium. As the interlayer distance increases, the sublattice magnetization gradually decreases to zero, becoming nonmagnetic when Δ*d*~35%. As the interlayer distance increases, the antiferromagnetic band gap decreases due to the reduction of staggered magnetization, and the spin-valley Chern number gradually approaches *C*_*sv*_~4, confirming that the non-quantized spin-valley Chern number is due to a large staggered magnetization. Next, we have investigated AB-stacked bilayer silicene nanoribbons with zigzag edges in order to confirm the corresponding spin-valley Hall effect. The ribbon width is represented by the number *N* of the C-C pairs on a layer in the unit cell.

**Figure 2:**
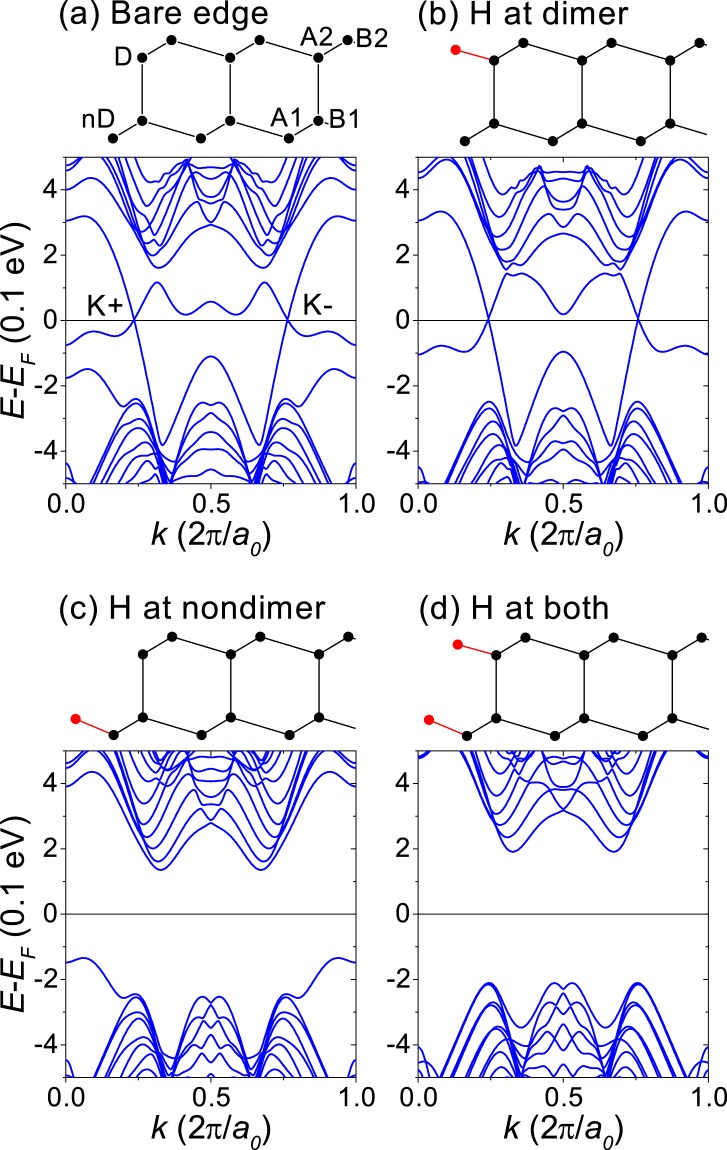
DFT band structures of zigzag-edge nanoribbons with *N* = 16. The top of each panel shows the corresponding edge structure, where red dots correspond to hydrogen. (**a**) Bare edges. (**b**) Hydrogen termination at the dimer edge sites. (**c**) Hydrogen termination at the nondimer edge sites. (**d**) Hydrogen termination at both edge sites. Energy bands with opposite spins were completely overlapped.

Figure 2 shows the band structures of the zigzag-edge nanoribbons with *N* = 16 near the Fermi level *E*_*F*_, where the energy bands with opposite spins were completely overlapped. As shown in Fig. 2(a), nanoribbons with bare edges have gapless edge states within the bulk gap, indicating that bilayer silicene can be a topologically nontrivial insulator while the spin-valley Chern number is not quantized. As can be seen in Fig. 2(b–d), there are gapless edge states when the dimer edge sites are terminated by hydrogen, but the gapless edge states disappear when the nondimer edge sites are terminated with hydrogen.

The gapless edge states in Fig. 2(a,b) are not connected with valence and conduction bands, which can be attributed to edge potentials. Nanoribbons inevitably have edge potentials, which can be induced by dangling *σ*-bonds, functional groups passivating the dangling bonds and edge-localized magnetic moments^25–27^. In a quantum valley Hall insulator, the dispersion of the edge state band is sensitive to edge potentials, varying from gapped flat-band to gapless chiral modes, because the edge potentials can introduce bearded-edge-like properties on the zigzag-edges^20^.

**Figure 3:**
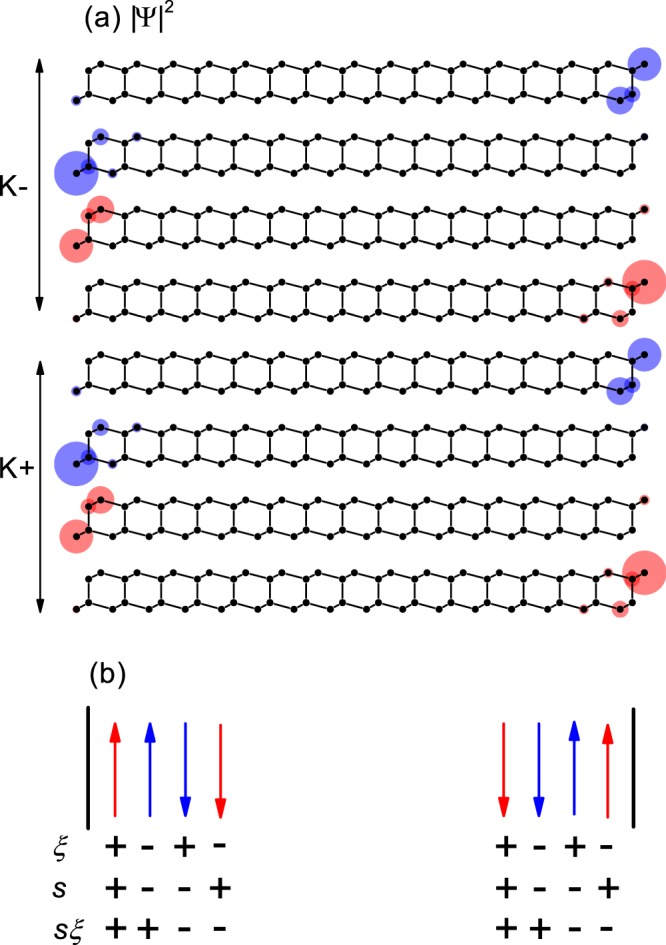
Schematics for gapless edge states in the zigzag-edge nanoribbons with bare edges. (**a**) The square of the wavefunction $|\Psi {|}^{2}$ at *E*_*F*_ for *N* = 16. (**b**) Schematics for the propagating states at *E*_*F*_. The red and blue colors represent the opposite spins. The up and down arrows correspond to the opposite propagating directions. *s*, $\xi$ and *s*$\xi$ are spin, valley and spin-valley indices, respectively.

To check whether the gapless edge states correspond to any quantum Hall effect, we have investigated the square of the wavefunction $|\Psi {|}^{2}$ near *E*_*F*_. Figure 3(a) shows $|\Psi {|}^{2}$ at *E*_*F*_ in the nanoribbons with bare edges, where the gapless edge states are confined on an edge, possibly indicating a quantum Hall effect. Figure 3(b) shows the schematics for the propagation of the gapless edge states constructed from $|\Psi {|}^{2}$ at *E*_*F*_ and the band velocity. Since each state contributes $\tfrac{{e}^{2}}{h}$ to the conductivity, the Hall conductivity can be estimated in units of $\tfrac{{e}^{2}}{h}$ by counting the number of states propagating in a given direction. In Fig. 3(b), we can obtain a spin-valley Hall conductivity *σ*_*sv*_ = 4 $\tfrac{{e}^{2}}{h}$ and can confirm that backscattering of the gapless edge states requires inversion of the spin-valley index *s*$\xi$. Time-reversal symmetry for a fixed valley and inter-valley separation for a fixed spin protects the gapless edge states from backscattering. As a result, the AB-stacked bilayer silicene is believed to be a quantum spin-valley Hall insulator, while the spin-valley Chern number is not quantized. In zigzag-edge nanoribbons with *N* = 32, we obtained the same results.

The gapless edge states with *σ*_*sv*_ = 4 $\tfrac{{e}^{2}}{h}$ correspond to *C*_*sv*_ = 4, which is consistent with the spin-valley Chern number obtained at the limits of small staggered magnetization shown in Fig. 1(f). For a quantum spin-valley Hall insulator, the bulk-edge correspondence is known to be exactly established only at a topological domain wall^4,18^. At a vacuum interface, the number of gapless edge states depends on the edge condition and may not correspond to the spin-valley Chern number. Nevertheless, the gapless edge states in Figs. 2 and 3 show that the AB-stacked bilayer silicene is a quantum spin-valley Hall insulator while the spin-valley Chern number is not quantized.

The (spin-)valley Chern number and the bulk-edge correspondence are based on the assumption that opposite valleys are well separated^19–23^. At an armchair edge, where opposite valleys are completely mixed, the bulk-edge correspondence has no meaning. For a large inversion symmetry-breaking potential, the Berry curvature still centered on each valley may be broadened and partially mixed with each other, and thus the (spin-)valley Chern number obtained by integrating the Berry curvature around each valley may deviate from the quantized value. To clarify the effect of partial valley mixing, the TB model of Eq. (1) for the AB-stacked honeycomb bilayer was investigated (see the Methods section).

**Figure 4:**
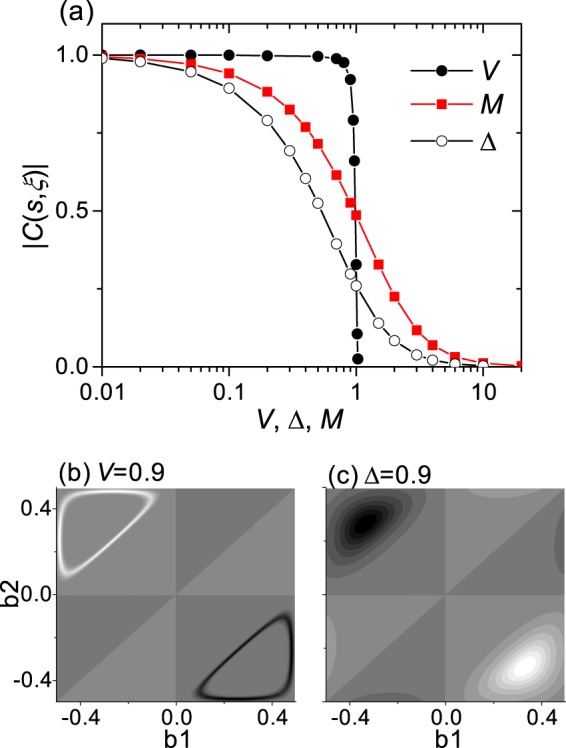
TB calculations for honeycomb bilayer sheet. (**a**) Absolute value of spin-valley-resolved Chern number $|C(s,\xi )|$ as a function of inversion symmetry breaking potentials. (**b**,**c**) Show the Berry curvature maps for *V* = 0.9 and Δ = 0.9, respectively.

Figure 4(a) shows the absolute value of spin-valley-resolved Chern number $|C(s,\xi )|$ as a function of the inversion symmetry-breaking potentials. We can consider two types of potential, interlayer potential difference 2*V* and staggered AB-sublattice potential Δ. For a small enough *V* and Δ, $|C(s,\xi )|=1$. While $|C(s,\xi )|$ abruptly changes from 1 to 0 as *V* increases to *t*_0_, $|C(s,\xi )|$ gradually decreases as Δ increases. In the intralayer ferrimagnetic and the interlayer antiferromagnetic order, the staggered magnetization *M*_*i*_ corresponds to a combination of a interlayer potential difference and a staggered AB-sublattice potential, with opposite signs for opposite spins. As *M* increases, $|C(s,\xi )|$ gradually decreases, too. The abrupt and gradual change of the spin-valley-resolved Chern number can be attributed to the Berry curvature.

Figure 4(b,c) show the Berry curvature maps for *V* = 0.9 and Δ = 0.9, respectively. For a small *V* and Δ, the Berry curvature is sharply peaked at each valley. The increase in *V* shifts the Berry curvature peak away from the valley while maintaining its sharp shape as shown in Fig. 4(b), and the mixing of the Berry curvature peak pertaining to each valley abruptly occurs when the Berry curvature peak reaches the Brillouin zone border. As shown in Fig. 4(c), the increase in Δ broadens and lowers the Berry curvature peak while maintaining its center at each valley, and the mixing of the tails of the Berry curvature peak pertaining to each valley gradually increases with Δ.

Under a staggered AB-sublattice potential Δ, the Berry curvature in a honeycomb monolayer was reported to be $\Omega (q)=3\Delta \xi /2{({\Delta }^{2}+3{q}^{2})}^{3/2}$ for small *q*, where *q* is the wavevector measured from the K point in the Brillouin zone^20^. As Δ increases, the Berry curvature peak broadens while maintaining its center on each valley, consistently with our results. Under an interlayer potential difference 2*V*, the Berry curvature in a honeycomb bilayer was reported to be $\Omega (q)=-\,2\xi {t}_{1}V{q}^{2}/{({q}^{4}+{t}_{1}^{2}{V}^{2})}^{3/2}$ for small *q*^23^. As *V* increases, the Berry curvature peak moves away from the valley, consistently with our results.

Regardless of the abrupt and gradual change of the spin-valley-resolved Chern number, the band gap increases as the inversion symmetry-breaking potential increases. It is clear that $|C(s,\xi )|=1$ corresponds to a quantum (spin-)valley Hall insulator with gapless edge states at a topological domain wall, and $|C(s,\xi )|=0$ corresponds to a topologically trivial insulator. To clarify whether a finite non-quantized $|C(s,\xi )|$ due to the partial valley mixing corresponds to a quantum (spin-)valley Hall effect, we investigated *N* = 80 zigzag-edge honeycomb bilayer nanoribbons with a kink-type topological domain wall.

**Figure 5:**
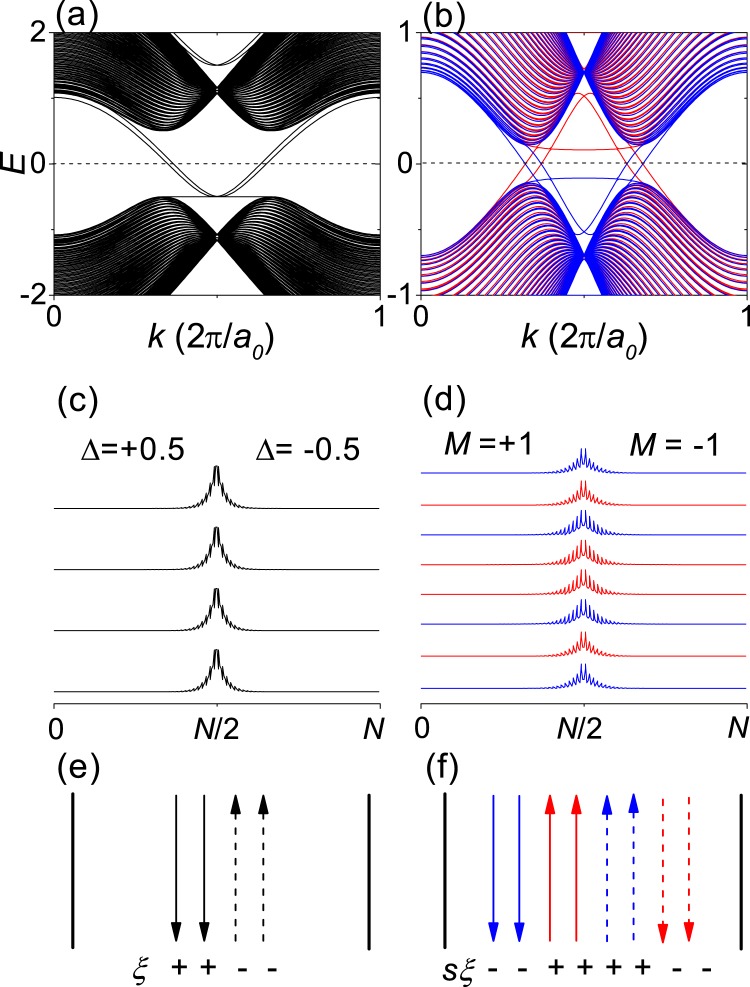
TB calculations for *N* = 80 zigzag-edge honeycomb bilayer nanoribbons with a kink-type topological domain wall. (**a**,**c**,**e**) Show the band structure, $|\Psi |$ at *E* = 0 and a schematic for the propagating states at *E* = 0, respectively, for Δ = ±0.5 in each half of the nanoribbon. (**b**,**d**,**f**) show the band structure, $|\Psi |$ at *E* = 0 and a schematic for the propagating states at *E* = 0, respectively, for *M* = ±1 in each half of the nanoribbon. The red and blue colors represent the opposite spins. The up and down arrows correspond to the opposite propagating directions. The solid and dashed arrows represent the opposite valleys.

Figure 5(a,c,e) show the band structure, $|\Psi |$ at *E* = 0 and a schematic for the propagating states at *E* = 0, respectively, for Δ = ±0.5 in each half of the nanoribbon. Figure 5(b,d,f) show the band structure, $|\Psi |$ at *E* = 0 and a schematic for the propagating states at *E* = 0, respectively, for *M* = ±1 in each half of the nanoribbon. In both cases, $C(s,\xi )\sim \pm \,0.5$ in each half of the nanoribbon. Within the bulk gap, we can see that there are gapless edge states which are well confined on the topological domain wall, and backscattering of the gapless edge states requires inversion of the (spin-)valley index.

According to the bulk-edge correspondence^4,21,22^, the number of gapless edge state per valley per spin at the topological domain wall should be equal to the difference of the spin-valley-resolved Chern number between the two topological domains, $C(s,\xi )-(\,-\,C(s,\xi ))=2C(s,\xi )$. In Fig. 5, the number of gapless edge state per valley per spin is 2, indicating that $C(s,\xi )$ should be 1 according to the bulk-edge correspondence. While the (spin-)valley Chern number obtained by integrating the Berry curvature around each valley deviates from the quantized value due to the partial valley mixing, the gapless edge states still correspond to the quantized (spin-)valley Chern number obtained at the limits of well-separated valleys if the opposite valleys are not completely mixed and so if the (spin-)valley Chern number is not too close to zero.

As shown in Fig. 5(a), there are gapless edge states corresponding to the quantum valley Hall effect if $\Delta \le 0.5{t}_{0}$. In the intralayer ferrimagnetic and interlayer antiferromagnetic order, the magnitude of the sublattice magnetization at the dimer sites (*M*_*D*_) is smaller than that at the nondimer sites (*M*_*ND*_). When ${M}_{D}={M}_{ND}$, there are gapless edge states corresponding to the quantum spin-valley Hall effect if ${M}_{ND}\le {t}_{0}$. As *M*_*D*_ decreases, the critical value of *M*_*ND*_ increases. When ${M}_{D}=0.1{M}_{ND}$, corresponding to Fig. 5(b), the gapless edge states survive if ${M}_{ND}\lesssim 3{t}_{0}$.

In the quantum (spin-)valley Hall effect with large staggered AB-sublattice potentials, the (spin-)valley Chern number is not well defined due to the valley mixing, but the gapless edge states still correspond to the (spin-)valley Chern number obtained at small potential limits. Even in the presence of spin mixing, the spin Chern number could be well defined by partitioning the occupied valence band into two spin sectors using the projected spin operator^28,29^. In a similar way, it is expected that the (spin-)valley Chern number can be well defined even in the presence of valley mixing, which requires further study.

To summarize, we have investigated AB-stacked bilayer silicene, known to be an antiferromagnetic insulator, by using the density functional theory calculations. While the spin-valley Chern number is not quantized, there exist backscattering-free gapless edge states within the bulk gap, indicating that the bilayer silicene is a quantum spin-valley Hall insulator. Using a tight-binding model for a honeycomb bilayer, we found that a non-quantized (spin-)valley Chern number may correspond to a quantum (spin-)valley Hall insulator under an inversion symmetry-breaking potential in the form of staggered AB-sublattice potential such as an antiferromagnetic order.

## Methods

A SIESTA package^30^ using a localized linear combination of numerical atomic-orbital basis sets was employed for the DFT calculations. A generalized gradient approximation of Perdew-Burke-Ernzerhof (GGA-PBE) was used for the exchange and correlation potential^31^. A double-$\zeta$ polarized basis set and norm-conserving Troullier-Martins pseudopotentials generated with a Perdew-Burke-Ernzerhof functional were used. The plane-wave cutoff energy of 350 Ry was used for the real-space grid, and *k*-points of 100 × 100 × 1 and 40 × 1 × 1 meshes in a Monkhorst-Pack scheme were used for bilayer silicene sheet and nanoribbons, respectively. The atomic coordinates were optimized by using the conjugated gradients method with a maximum force tolerance of 0.1 eV/nm. The equilibrium lattice constant *a*_0_ = 0.386 nm and the interlayer distance *d*_0_ = 0.251 nm between the covalently-bonded dimer sites were obtained from the total energy minimum, in agreement with a previous work^14^. Zigzag-edge nanoribbons were considered as a one-dimensional system periodic in a zigzag direction. The ribbon width is represented by the number *N* of the C-C pairs on a layer in the unit cell. The spin-orbit coupling, much smaller than the antiferromagnetic gap, was neglected.

Using the wavefunctions obtained from the DFT calculations on the bilayer silicene sheet, maximally localized Wannier functions were constructed within the Wannier90 code^32^. Berry curvatures were calculated based on the Wannier interpolation. Using the anomalous Hall conductivity calculation routine implemented in Wannier90 code, the spin-valley-resolved Chern number $C(s,\xi )$ with spin *s* and valley $\xi$ was calculated by integrating the Berry curvature over a half Brillouin zone. The spin-valley Chern number was defined as ${C}_{sv}=C(\,\uparrow ,{\rm{K}}\,+\,)-$ ${\rm{C}}(\,\downarrow \,,{\rm{K}}\,+\,)-{\rm{C}}(\,\uparrow \,,{\rm{K}}\,-\,)+{\rm{C}}(\downarrow \,,{\rm{K}}\,-\,)$ and the spin-valley Hall conductivity can be expressed as ${\sigma }_{sv}={C}_{sv}$ $\tfrac{{e}^{2}}{h}$^33^.

A *π*-band TB model Hamiltonian with nearest-neighbor hopping was introduced to model the AB-stacked honeycomb bilayer with broken inversion symmetry^18,34^:

1 $$H=-\,\sum _{\langle i,j\rangle s}\,{t}_{i,j}{c}_{is}^{\dagger }{c}_{js}+\sum _{is}\,({V}_{i}+{\Delta }_{i}+s{M}_{i}){c}_{is}^{\dagger }{c}_{is}.$$

The hopping amplitude ${t}_{i,j}={t}_{0}$ for in-plane hopping and ${t}_{i,j}={t}_{1}$ for interalyer hopping between the dimer sites. We set *t*_0_ = 1 and ${t}_{1}=0.1$. The second term corresponds to on-site potentials breaking inversion symmetry. The AB-stacked honeycomb bilayer has four sites A1, B1, A2 and B2 as shown in Fig. 2(a). On the basis of (A1,B1,A2,B2), *V*_*i*_ = (−1, −1, +1, +1)*V* corresponds to the interlayer potential difference. Δ_*i*_ = (−1, +1, −1, +1)Δ corresponds to the staggered AB-sublattice potential. *M*_*i*_ = (+1, −0.1, +0.1, −1)*M* models the intralayer ferrimagnetic and the interlayer antiferromagnetic order of AB-stacked bilayer silicene with a spin *s* = ±1.
